# CT based 3D reconstruction of the forefoot’s blood supply in a white rhinoceros

**DOI:** 10.1186/s13028-024-00732-2

**Published:** 2024-03-07

**Authors:** László Zoltán Reinitz, Claudia Cerny, Endre Papp, Alexandra Biácsi, Daniel Fajtai, Örs Petneházy

**Affiliations:** 1 Department of Anatomy and Histology, University of Veterinary Medicine Budapest, István utca 2, Budapest, H-1078 Hungary; 2 Nyíregyházi Állatpark Nonprofit Kft. (Sosto Zoo), HRSz15010/2, Sóstói út, Nyíregyháza, H-4431 Hungary; 3 Medicopus Nonprofit Kft, Tallián Gy u. 20-32, Kaposvár, H-7400 Hungary; 4 Department of Physiology and Animal Health, Institute of Physiology and Nutrition, Hungarian University of Agriculture and Life Sciences, Kaposvár Campus, Guba Sandor u. 40, Kaposvár, H-7400 Hungary

**Keywords:** 3D model, *Arcus palmaris profundus*, *Ceratotherium simum*, Deep palmar arch, Digital cushion, Vascular anatomy

## Abstract

**Background:**

The white rhinoceros (*Ceratotherium simum*) is close to extinction, listed as “Near Threatened”, with a decreasing population on the Red List of Threatened Species of the International Union for Conservation of Nature. In at least 50% of the specimens in captivity, podiatric diseases, such as osteitis, osteomyelitis, chip fractures, enthesophytes, fractures and osteoarthritis were found during necropsy. These osteal deformations cause further pathogenic alterations in the soft tissues, particularly in the digital cushion. The literature provides good description of the skeleton of the rhino’s limbs, but similar for the vascular system is non-existent. In order to recognize the symptoms in an early state and for a successful surgical treatment, precise knowledge of the vascular anatomy is essential. The purpose of our study was to provide detailed anatomical description of the blood supply of the digits and that of the digital cushion.

**Results:**

The blood supply of the distal foot, digits and digital cushions were perfectly visible on the reconstructed and coloured 3D models. The deep palmar arch provided not only the blood supply to the digits but had a palmaro-distal running branch which developed a trifurcation proximal to the proximal sesamoid bones of the third digit. Two of its branches participated in the blood supply of the digits’ proximal palmar surface, while the major branch supplied the digital cushion from proximal direction.

**Conclusions:**

Our findings show a unique blood supply: the main vessels of the digital cushion stem both directly from the deep palmar arch and from the digits’ own arteries. The detailed description of vessels may be useful in planning surgery of the region and also in cases where the veins of the ear are not accessible.

**Supplementary Information:**

The online version contains supplementary material available at 10.1186/s13028-024-00732-2.

## Background

The white rhinoceros (*Ceratotherium simum*) is one of the largest terrestrial mammals, with its weight averaging 2.3 tons [1]. Due to reduction of its natural habitat and intensive poaching the species is classified as “Near Threatened” on the Red List of Threatened Species of the International Union for Conservation of Nature [2, 3]. Due its size, its limbs play a prominent role in the health status of the individual, as even a small decline in the condition of any limb can result in limited freedom of movement, thus decline in the amount of consumed food, consequent decline in general health condition, fertility and, eventually, death [1, 2, 4, 5]. A postmortem study found osteopathy on at least one leg in 81.5% of 27 examined rhinos [2]. Bone tissue lesions, such as osteitis, osteomyelitis, chip fractures, enthesophytes, fractures and, in more than 50% of the cases, osteoarthritis are usually the causes of pathogenic soft tissue alterations. In more than 60% of the studied cases, osteoarthritis affected the distal interphalangeal joint [2, 6–8]. The most common soft tissue alterations are fissure of the horn wall, ulceration and lesion of the digital cushion or sole, pododermatitis, abscess or laminitis [5–8]. Disease detection and early diagnosis is made more difficult by the fact that, as prey animals, they are excellent at hiding symptoms until they reach a severe state [5–8], and diagnosis is often only possible postmortem [9]. Studies of extinct species show that their ancestors suffered from the same osteopathological problems, suggesting that the wild population is probably also affected [10]. Despite its high prevalence, reports on osteoarthritic conditions are scarce [11, 12], partly due to the difficulties associated with diagnostics in these mega-herbivores [9]. Prognosis of conservative, non-sedated treatment of these diseases is poor; conservative treatment of the affected area under anaesthesia is the most commonly elected procedure, despite mixed results [12, 13]. Surgical solutions such as amputation of the affected digit are rare [14–16], partially due to the limited anatomical information available about the region.

The limbs of the white rhinoceros are stout, graviportal-type extremities, that are both capable of carrying large weight and are adapted for running [17, 18]. The forelimbs carry 60% of the weight of the animal and decelerate the forward movement of the body [19]. The *radius* and the *ulna* are separated, but are tightly connected, both being short and wide. The carpus has a standard layout with 8 carpal bones in 2 horizontal rows (proximal row: *os carpi radiale, os carpi intermedium, os carpi ulnare, os carpi accessorium*, distal row: *os carpale I* (CI), *II* (CII), *III* (CIII), *IV* (CIV)) [8].

CI does not have a distal joint surface. CII and CIII connect to the corresponding metacarpal bones (*os metacarpale II (McII) & os metacarpale III* (McIII)). CIV is connected to both McIII and the fourth and fifth metacarpal bones (*os metacarpale IV* (McIV) *& os metacarpale* (McV)). Of all the metacarpal bones, the centrally located McIII is the thickest. McII and McIV are similar in their diameter to McIII but are slightly shorter, while McV is only rudimentary; it is less than one third of the length of McIV. Rhinoceroses have three digits on each forelimb (DII, DIII and DIV), of which the central one (DIII) is the most prominent [2, 6]. Each digit bears 2 proximal sesamoid bones to support the metacarpophalangeal joint, but neither has distal sesamoid bones [2, 20] except in the Indian rhinoceros (*Rhinoceros unicornis*) [21]. The *phalanx distalis* in both DII and DIV is asymmetric, with a single palmar process projecting in the abaxial direction; the corresponding hoof capsules are drop-shaped. The *phalanx distalis* of DIII is symmetric, it has 2 palmar processes, both projecting in the abaxial direction, with its hoof capsule being semi-circular [5, 8, 22].

The vertical axis of the brachium is directed medially up to the carpal joint, where it breaks and becomes vertical [9]. The feet are semi-digitigrade, thus, the digits make an angle of approximately 45 degrees with the ground, which results in a single, large, fibroelastic digital cushion (*torus digitalis*) between the sole and the phalanges [23]. The axis runs in the centre of digit III, making it the primary bearer of the weight [20]. The sole is rounded & almost symmetrical [6].

There is no available histological description of the rhinoceros’ digital cushion, but that of the elephant is frequently mentioned in the literature [24, 25]. A recent study on the elephant’s digital cushion [23] concluded that the micromorphology of the elephant’s digital cushion has strong similarities to that of the human foot pad [26] and the digital cushion of cattle [27] but it is largely different from the equine digital cushion [28]. Cattle, horses and rhinoceros are all members of the clade or grand order Euungulata (true ungulates) but fall into two different orders (Artiodactyla or even-toed ungulates (cattle) and Perissodactyla or odd-toed ungulates (horses and rhinoceros)). Elephants fall under the clade or grand order of Peanungulates (sub-ungulates), in the order of Proboscidea. Therefore, the structure of their digital cushion is based on the static and dynamic mechanics of these species, as the equine digital cushion absorbs more dynamic loads, which are also different in their timings and magnitude [29]. Because the movement of the rhinoceros is much closer to that of the similarly graviportal elephant than to that of the horse [1, 6], it presumably has the digital cushion microstructure closer to that of the elephant despite being evolutionarily much closer to the horse.

There is no available description or depiction of the blood supply of the rhinoceros’ distal forefoot. In domestic mammals (dog, cat, equines, cattle, sheep, goat, swine) the deep palmar arch (*arcus palmaris profundus*) gives rise to the metacarpal arteries, which anastomose with the terminal portion of the median artery (*a. mediana*) to supply the digits from the palmar side via the common digital arteries (*aa. digitales communes*). These latter run between the digits and supply the palmar surfaces of the adjacent digits, while a much weaker dorsal blood supply originates from the cranial superficial antebrachial artery (*a. antebrachialis superficialis cranialis*) and the dorsal carpal plexus (*rete carpi dorsale*) through the dorsal metacarpal arteries (*aa. metacarpeae dorsales*). The dorsal sides of the digits are also supported through small branches originating from the palmar system (*rr. dorsales phalangis*). Each weight-bearing digit has its own digital cushion. Their supplying vessels (*rr. tori digitales*) are paired for each digit, discharging distal to the proximal interphalangeal joints [29, 30]. In elephants the terminal portion of the deep palmar arch runs around the distal end of McV from its dorsal aspect, and the metacarpal arteries continue directly into the corresponding common digital arteries. The deep palmar arch also discharges a short trunk close to the height of the carpometacarpal joints, which develops a trifurcation. One of its branches supplies the prepollex, one is directed dorso-distally, the third distally. The latter two vessels both run in approximately the median plane of the distal limb, supplying the digital cushion [31].

Most anatomical descriptions of rhinoceroses are based on traditional anatomical techniques [1, 19, 21]. One research group performed detailed Computed Tomography (CT) on the feet which produced high-quality images of the skeleton, but the main focus of the studies was to create radiographic standards for clinical examinations and to facilitate the early diagnosis of subclinical morphological lesions rather than to provide anatomical descriptions [7, 8].

In this study we aimed to define the blood supply of the rhinoceros’ forelimb foot, clarifying the arteries of the digital cushion, and to define the course of the digital arteries. Such results may be used in planning surgeries in affected regions to improve the recovery time and the overall safety of the procedure, can help in understanding the pathophysiology of foot related problems and may influence the treatment of a lesion in the digital cushion.

The left forelimb of a white rhino, which was kept in a zoo and died due to causes unrelated to its limbs, was examined with native, thin-slice Computed Tomography (CT). Subsequently, barium sulphate-containing contrast medium was injected through a catheter into the median artery. This was followed by repetition of the CT examination with the limb being held in the same position. The sequences were processed with 3D Slicer using semi-automatic segmentation methods.

## Methods

### Specimen acquisition

The Nyiregyháza Állatpark Nonprofit Kft (Sosto Zoo; HRSz15010/2, Sóstói út, H-4431 Nyíregyháza, Hungary) offered the cadaveric left forelimb of a deceased 7-year-old female white rhinoceros (*Ceratotherium simum simum*) to the Department of Anatomy and Histology of the Budapest University of Veterinary Medicine (István utca 2, H-1078 Budapest, Hungary) for anatomical research. The CITES registration number of the specimen was HU/FTV/15120-1/2006 and the transponder number was 456B503030. The animal was born in captivity, had no history of limb or circulatory diseases and died of malignant liver tumor. The limb was severed at the *art. mediocarpea* and was stored in a -4 °C environment.

### Image acquisition

The limb was transported to the Kaposi Mór Somogy County Teaching Hospital, c/o Dr. József Baka Diagnostic, Radiation Oncology, Research and Teaching Center (Guba Sándor utca 40, H-7400 Kaposvár, Hungary) in a transport container with an inner temperature of -4 °C. Following defrosting the surfaces were hydrated and an elastic cannula with a diameter corresponding to the size of the artery was inserted into the *a. mediana.* The limb was placed in a plastic tube which secured its position but allowed access to the cannula. Overall, three separate CT scanning series were completed with identical settings (transverse slices; caudal vision of image; 140 kV; 240 mAs; 0.6 mm slide thickness; 492 × 492 mm Field of View with isotropic voxels; reconstruction kernel: T80f) using a Siemens Somatom Sensation Cardiac CT (multislice scanner, Siemens AG, Erlangen, Germany) which resulted in a total of 1833 images. The first series was processed without any contrast medium. After that, 20 ml of Micropaque® (Guerbet, Bruxelles, Belgium), a barium-sulphate containing oral contrast medium was injected into the cannula, and the scanning was repeated. This was followed by injecting a further 28 ml of the same contrast medium into the cannula and performing another CT series.

### Visualisation

**Fig. 1:**
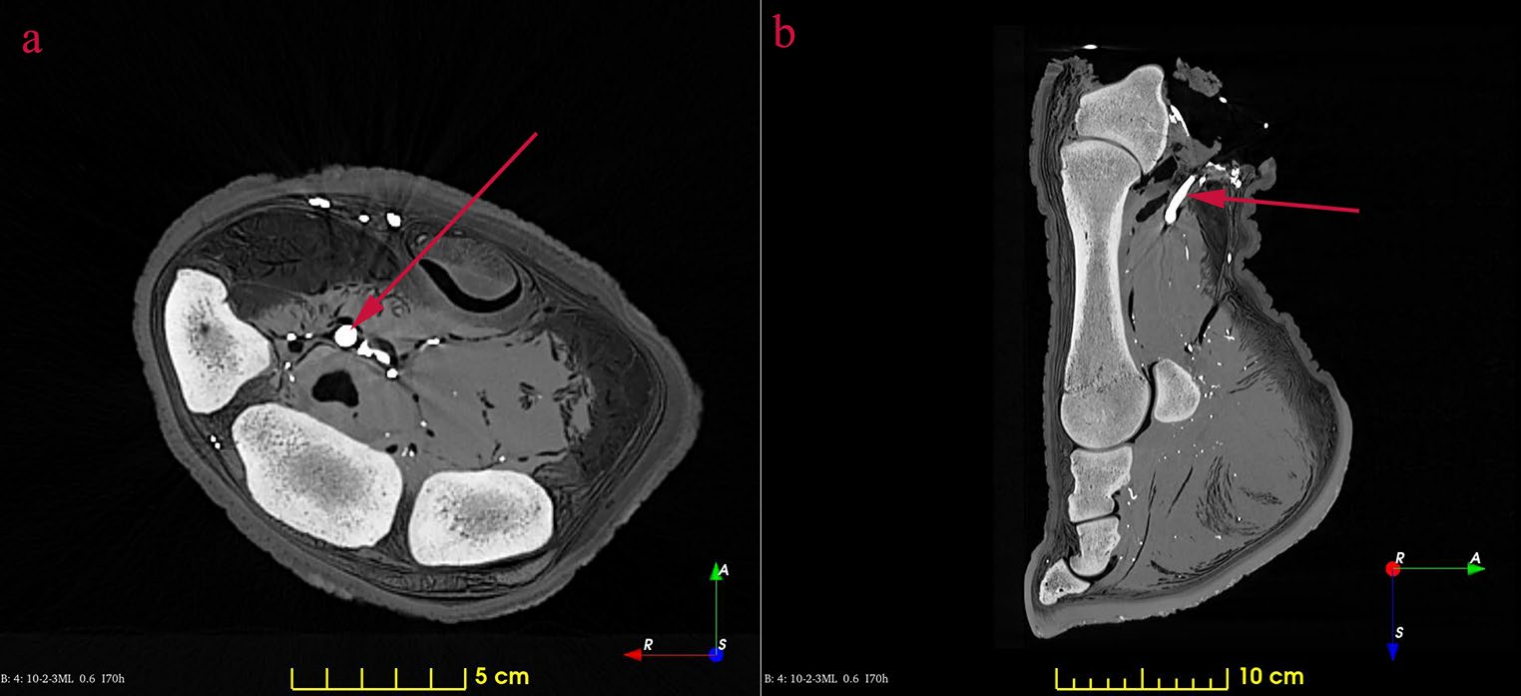
Contrast-filled CT images of the rhinoceros left forefoot. The red arrows show the *a. mediana*. **a**: horizontal plane; **b**: sagittal plane

After a thorough evaluation of the datasets by a clinical veterinarian no pathological lesions were identified. It was concluded that the vessels were significantly better traceable on the third series than on the second; therefore, the first series was used for the bones and the third series for the vessels (Fig. 1).

The selected data sets were processed in the 3D Slicer program, version 4.11. 3D Slicer is a free software platform, designed for medical image processing and visualisation; its open-source mainframe was developed with the support of the National Institutes of Health, and it receives significant contribution from a worldwide community of developers [32, 33].

**Fig. 2:**
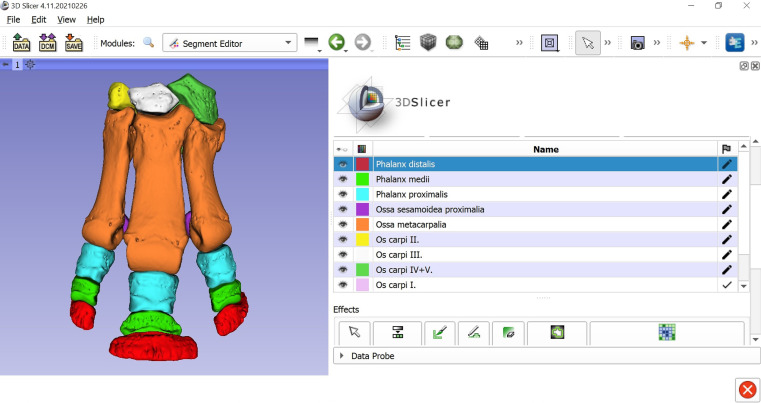
Screenshot of 3D Slicer during the segmentation of the skeleton of the rhinoceros left forefoot

Bone structures were segmented in automatic mode using the “Threshold” effect of the “Segment Editor” module which recognizes structures of the same intensity range, highlights them in any preferred colour and generates a 3D model based on the selected voxels. Each slice was manually analysed by a veterinary anatomist in order to verify the selections. A new “Segment” with an associated colour was assigned to each group of bones (each bone of the distal carpal row, the metacarpals, the proximal sesamoid bones and the proximal, middle and distal phalanges) in order to differentiate them (Fig. 2).

The vessels were reconstructed with the “Subtract scalar volumes” module. The first and the third sequences were loaded simultaneously, and the module recognized and subtracted the differences between them, which was the vessels filled with the contrast medium. The small, terminal branches were removed manually from every slice. The 3D model of the vessels was then created using the Segment Editor module in a similar fashion as it was used for the bones.

Finally, the 3D models of the vessels and the skeleton were merged into one file.

## Results

The limb was examined distal to the mediocarpal joint (art. mediocarpea). All anatomical expressions are based either on the existing literature of the rhinoceros [1, 5, 7, 8, 21] or on the homologous structures of domestic mammals presented in the Veterinary Anatomical Nomenclature [30] and major veterinary anatomy books [22, 29], unless otherwise indicated in the description.

**Fig. 3:**
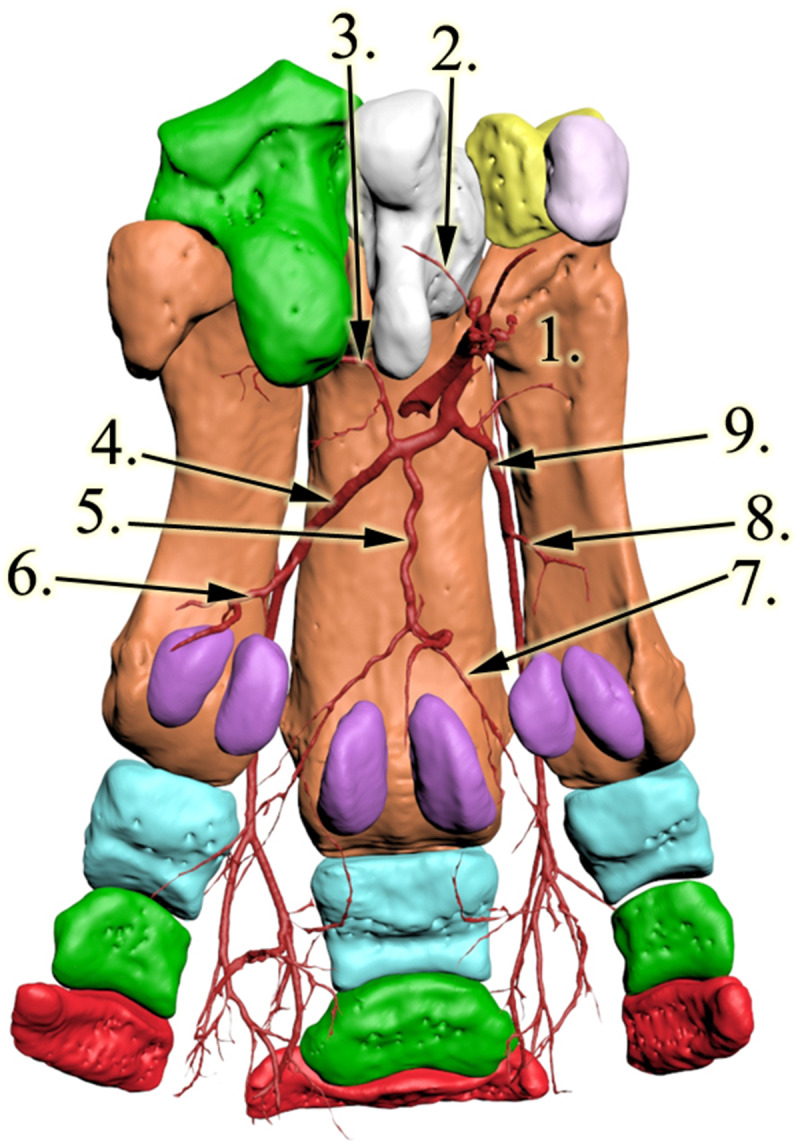
3D reconstruction of the rhinoceros left forefoot skeleton and blood supply, palmar view. 1: os metacarpalia II, 2: a.radialis, 3: a. interossea caudalis, 4: arcus palmaris profundus, 5, newly found vessel, 6: a. metacarpea palmaris profundus IV, 7: medial branch of the newly found vessel, 8: a. metacarpea palmaris profundus II, 9: a.metacarpea palmaris II

All bones were identified as expected based on the literature, with four bones forming the distal row, four metacarpal bones present, and each of the three digits had 3 phalanges and a pair of proximal sesamoid bones (Fig. 3).

**Fig. 4:**
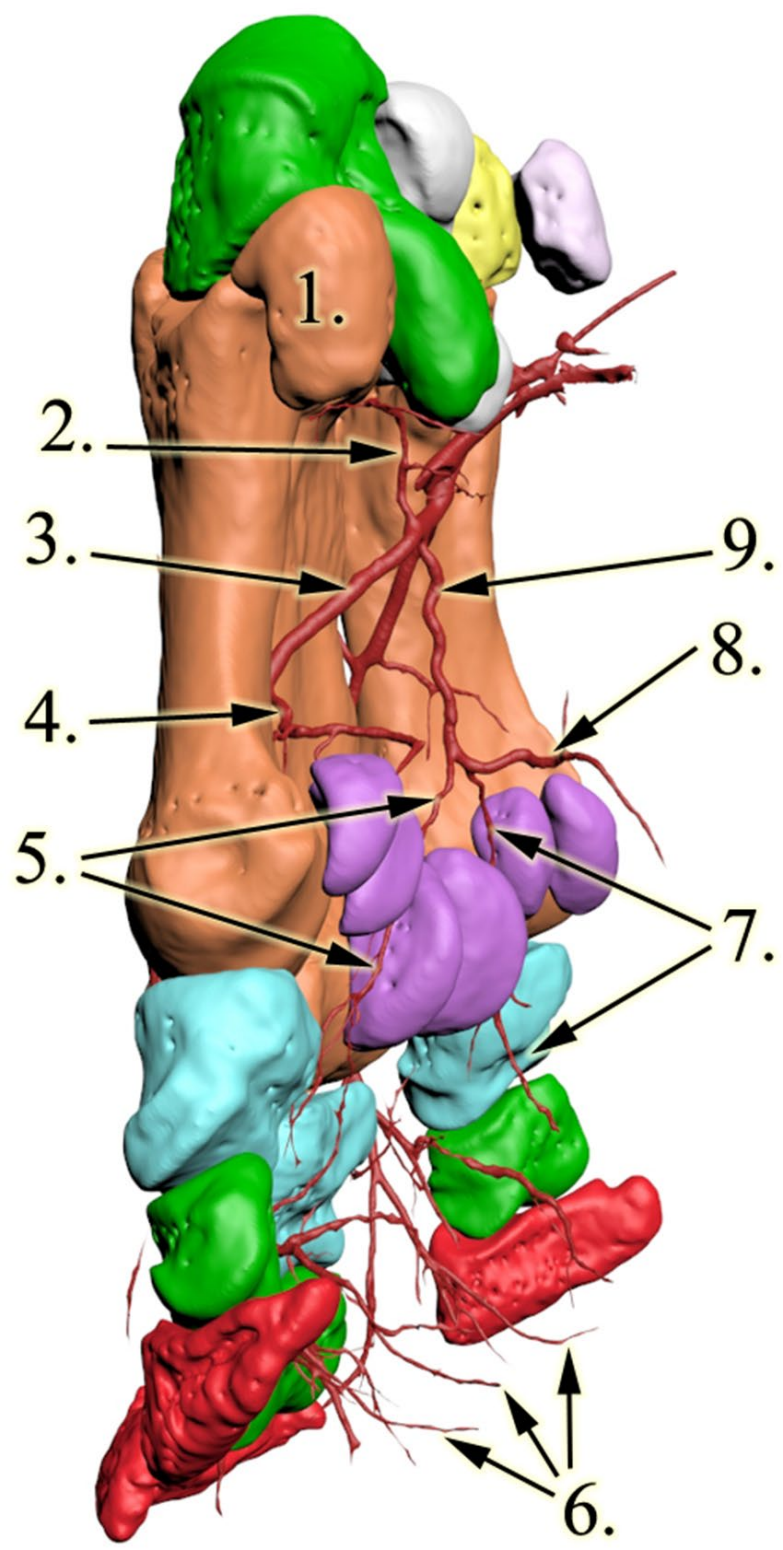
3D reconstruction of the rhinoceros left forefoot skeleton and blood supply, latero-palmar view. 1: os metacarpalia V, 2: a. interossea caudalis, 3: arcus palmaris profundus, 4: a. metacarpea palmaris profundus IV, 5: lateral branch of the newly found vessel, 6: rr. tori digitales, 7: medial branch of the newly found vessel, 8: palmar branch of the newly found vessel, 9: newly found vessel

**Fig. 5:**
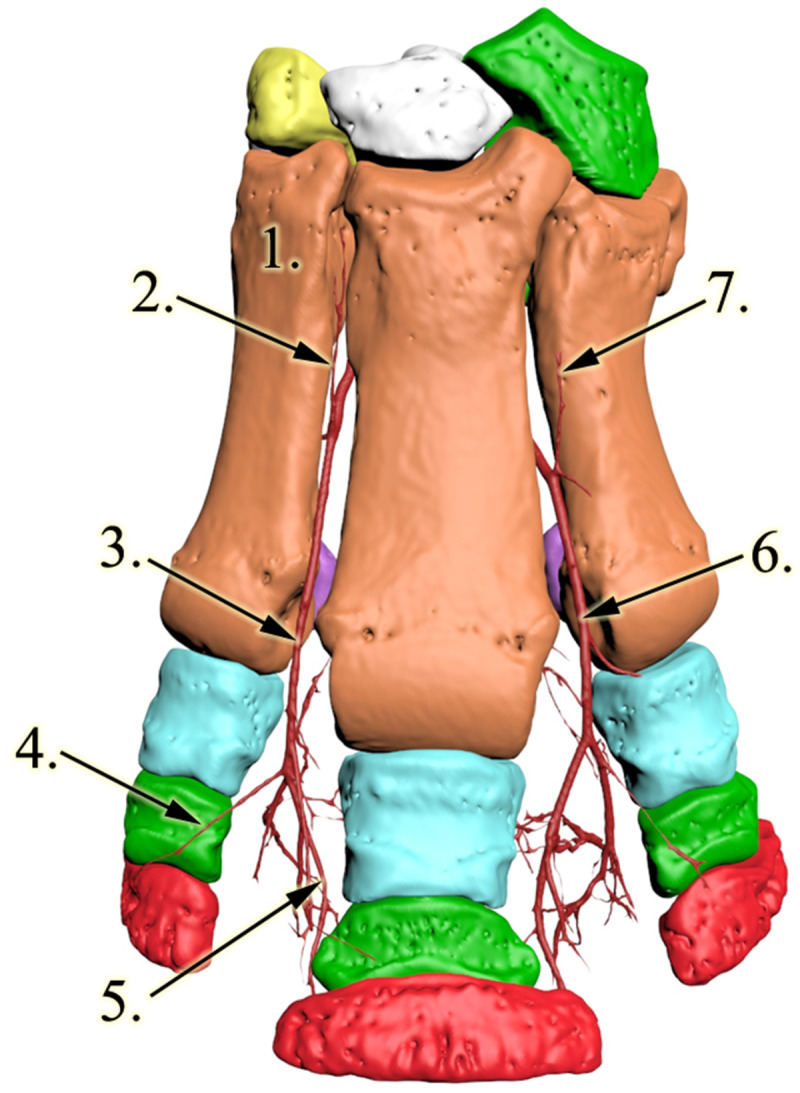
3D reconstruction of the rhinoceros left forefoot skeleton and blood supply, dorsal view. 1: os metacarpalia II, 2: a. metacarpea dorsalis II, 3: a. digitalis dorsalis communis II 4: a. digitalis dorsalis proprius II axialis, 5: a. digitalis dorsalis proprius III medialis, 6: a. digitalis dorsalis communis III, 7: a. metacarpea dorsalis III.

The carpal section of the median artery was damaged, and some contrast media got out of the vessel and contaminated this region. The *a. mediana* was the major vessel for the deep palmar arch which begun distal to the medial aspect of the carpometacarpal joint. Two further, smaller vessels were found to join the arch from the proximal direction: the medial was the *a. radialis*, which descended between CII and CIII; the lateral was the *a. interossea caudalis* which descended between CIV and McV. The latter had a small horizontal section under CIV, crossed the lateral aspect of CIII and then turned vertical towards the deep palmar arch. The arch ran close to the metacarpal bones in a disto-palmar direction until it reached the axial proximal sesamoid bone of McIV. The arch first dispatched the *a. metacarpea palmaris II*, which ran between McII and McIII in a distal direction to the dorsal side (Figs. 3 and 4; Additional file 1). After running through the interosseal space, it became the *a. digitalis dorsalis communis II*, which continued in branches coursing on both the dorsal and palmar surfaces of DII (*a. digitalis dorsalis et palmaris proprius II. axialis*), as well as the medial surface of DIII (*a. digitalis dorsalis et palmaris proprius III. medialis*) Fig. 5. While these vessels were responsible for supplying the entire dorsal surface of DII and the entire medial dorsal surface of DIII, the palmar branches returned to the palmar side only at the height of the proximal interphalangeal joint, thereby supplied only the distal portion of the digits’ palmar side. The *a. metacarpea palmaris II* also had a branch (*a. metacarpea palmaris profundus II*), before it penetrated the interosseal space, which supplied the palmar side of McII and reached the corresponding proximal sesamoid bones. A similar branch (*a. metacarpea palmaris profundus IV*), which supplied the palmar aspect and the proximal sesamoid bones of McIV, was the last branch of the deep palmar arch (Figs. 3 and 4). After it was dispatched, the arch became the *a. metacarpea palmaris III*, which ran parallel to the *a. metacarpea palmaris II* described earlier: it supplied the entire dorsal and the distal palmar surfaces of DIV (*a. digitalis dorsalis et palmaris proprius IV. axialis*), and the lateral surfaces of DIII (*a. digitalis dorsalis et palmaris proprius III. lateralis).* The *aa. metacarpeae palmares II* and *III* both received a supporting branch on the dorsal side from the proximal direction (*a. metacarpea dorsalis II et III*).

The deep palmar arch had a branch which did not have a matching homologue in the domestic mammals. This vessel started at the proximal third of McIII and ran in the vertical median axis of the foot with small bends, close to the bone’s palmar surface, 3–4 cm away from the skeleton. As it reached the height of the proximal sesamoid bones of McIII it developed a trifurcation. One branch ran medially and supplied the proximal palmar sides of DIII from the medial side and the full proximal palmar side of DII (*a. digitalis palmaris communis II*). The second vessel had a symmetric path in the lateral direction; thus it supplied the proximal palmar side of DIII from the lateral side and the full proximal palmar side of DIV (*a. digitalis palmaris communis III*). The third vessel ran in the palmar direction and turned distally to supply the digital cushion. The digital cushion received further vessels that were dispatched from every palmar proper artery described earlier at the level of the corresponding proximal interphalangeal joints (Figs. 3 and 4; Additional file 1).

## Discussion

Reconstructive anatomical techniques, like those used in this study, provide much more detailed assessment and much better visualisation then traditional methods [34, 35]. In our case, as the specimen was frozen for a long period of time, it significantly dried, which would have made dissection very difficult; the CT-based reconstruction by comparison offered much better visualisation. Of the vertical running arteries of the region which we expected to find, only the *a. mediana* was located. The contrast medium penetrated into the others (*a. interossea caudalis, a. radialis, aa. metacarpeae dorsales II et III*) as well, but did not return to the cut surface, indicating that these smaller vessels were damaged during the removal of the limb. Based on the images, sufficient contrast agent was added to highlight the major, clinically important vessels, without unnecessarily overfilling the small capillary vessels which could obscure them. Excess contrast agent can result in beam hardening artefacts [36] which can compromise visualisation of nearby regions. The contrast agent clearly reached the digital cushion (Figs. 1 and 4/6) and the peripheral regions (Figs. 4 and 5).

In our specimen the blood supply of the forefoot was significantly different from that of domestic mammals or the elephant. In these latter species the palmar and dorsal sides of the foot have dedicated vessels, with the palmar side being more dominant; some of the palmar vessels also have dorsal branches interconnecting at the sides. In the rhinoceros the deep palmar arch supplies the dorsal side as well, as the palmar metacarpal arteries run through their corresponding interosseal spaces. In a unique arrangement, which has not been observed in any other species, these arteries return to the palmar side to supply the distal portion of the digit. As a result, the blood supply of the distal palmar metacarpal region and the palmar side of the proximal phalanges is weaker than in other species. To compensate for this, a small, independent branch supplies this region on both the medial and the lateral sides, running towards the proximal sesamoid bones of McII and McIV respectively (*a. metacarpea palmaris profundus II et IV*). On the disto-palmar side of McIII a truncus is visible originating from the deep palmar arch which terminates in trifurcation. Two of its subsidiary vessels support the palmar aspect of McIII and that of the proximal phalanges of the digits, further compensating for the lack of palmar digital branches in this area. The third branch of the tripus supplies the digital cushion. Elephants also have a trunk from the deep palmar arch with a trifurcation in this region, but that is much shorter [31], with one branch supplying the prepollex and the other two the digital cushion. Hence, the blood supply of the digital cushion in the rhinoceros is also unique. Paired vessels in the direction of the digital cushion, originating from each digits’ own palmar arteries are visible, just like in the domestic ungulates (equines, cattle, sheep, goat, swine; *rr. tori digitales*) whilst the largest supplying vessel, the terminal branch of the trifurcation, supplies it from the proximal direction, resembling that in the elephant. The weaker direct blood supply of the metacarpophalangeal region when compared to the many alternatives for the interphalangeal joints supports the theory that in rhinoceroses these joints have a larger weight-bearing role than the metacarpophalangeal joint [2, 6–8], which is the prominent joint in domestic ungulates and elephants [29, 37].

In their movement rhinoceroses show similarities with both elephants, due to their extreme size, and the domestic ungulates, being able to canter and gallop [2, 6, 18]. This is probably the reason for having a single digital cushion, as in the elephant, but its blood supply shows similarities with domesticated species as well. The digital cushion itself may have a unique microstructure that requires further micromorphological study, as it is not consistent with the described structures of the distal limb of any other species. In domestic ungulates the branches of the *rete carpi dorsale* (*aa. metacarpeae dorsales*) can be found in the dorsal region. Whilst we were able to confirm the presence of two dorsal metacarpal arteries, we could not provide information regarding the *rete carpi dorsale* due to the distal separation of the foot.

Anatomical descriptions of rare, endangered species are based on a limited number of specimens. Often only one [37–39] or two [40] animals are examined; having four or more subjects involved in a study is only possible with a larger captive population [41–43]. Having one specimen to study is a clear limitation, but earlier studies indicated that rhinoceroses have no tendency to develop significant individual anatomical variations in this region [1, 7, 8], therefore our results are likely anatomically reproducible and could be used as a basis for further studies.

## Conclusions

As our single subject was free of foot diseases or any abnormalities in the forelimb, the created model and description of the blood supply is deemed most likely anatomically correct for all examples of the species. The anatomy of the other four rhinoceros species is largely similar, except for the presence of the distal sesamoid bone in the great white rhinoceros; therefore we would expect that this blood supply is found in the other species as well.

The main blood vessel of the digital cushion originates from the newly discovered trunk of the deep palmar arch, reaches it from the proximal direction, supported by vessels from the palmar proper arteries of the digits. Anatomically, this long trunk is comparable with the similar trunk in the elephant, because they have similar origins and both play a crucial role in the blood supply of the digital cushion, although the rhino’s trunk has more elevated role in the blood supply of the metacarpophalangeal joints. Accordingly, we suggest a similar name (“*a. tori digitalis communis*”), while its branches should be named according to the supplied region (“*r. phalangis proximalis medialis, r. phalangis proximalis lateralis*; *a. tori digitalis proximalis*).

The blood supply of the digits happens almost exclusively from the palmar side.

Our research is the first such description of the blood supply of the rhinoceros’ forefoot blood supply and it revealed a unique structure. The animations and 3D printable models provide a great opportunity for both education, surgical planning, understanding the development of lesions on the sole and the digital cushion, and can provide options for emergency vessels.

### Electronic supplementary material

Below is the link to the electronic supplementary material.

**Additional file 1**: Animation of the skeleton and arteries of the rhinoceros’s left forefoot, uncompressed AVI video file. Red bones: phalanx distalis; light green bones: phalanx medii; light blue bones: phalanx proximalis; dark purple bones: os sesamoidea proximalis; orange bones: os metacarpalia II-V; light purple bone: os carpi I, yellow bone: os carpi II, white bone: os carpi III, dark green bones: os carpi IV, dark red: vessels; blue vessel: newly identified trunk

## Data Availability

Data supporting reported results can be found here: 10.6084/m9.figshare.23054258.

## Declarations

### Prior publication

Data have not been published previously.
